# The Involvement of Neuroinflammation in the Onset and Progression of Parkinson’s Disease

**DOI:** 10.3390/ijms241914582

**Published:** 2023-09-26

**Authors:** Anamaria Jurcau, Felicia Liana Andronie-Cioara, Delia Carmen Nistor-Cseppento, Nicoleta Pascalau, Marius Rus, Elisabeta Vasca, Maria Carolina Jurcau

**Affiliations:** 1 Department of Psycho-Neuroscience and Rehabilitation, Faculty of Medicine and Pharmacy, University of Oradea, 410073 Oradea, Romania; anamaria.jurcau@didactic.uoradea.ro (A.J.); dcseppento@uoradea.ro (D.C.N.-C.);; 2 Department of Medical Disciplines, Faculty of Medicine and Pharmacy, University of Oradea, 410087 Oradea, Romania; 3 Department of Oral Rehabilitation, Faculty of Medicine “Vasile Goldis” Arad, 310025 Arad, Romania; 4 Faculty of Medicine and Pharmacy, University of Oradea, 410073 Oradea, Romania

**Keywords:** Parkinson’s disease, neuroinflammation, microglia, α-synuclein, M1 phenotype, M2 phenotype, astroglia, gut dysbiosis, signaling pathways, therapy

## Abstract

Parkinson’s disease is a neurodegenerative disease exhibiting the fastest growth in incidence in recent years. As with most neurodegenerative diseases, the pathophysiology is incompletely elucidated, but compelling evidence implicates inflammation, both in the central nervous system and in the periphery, in the initiation and progression of the disease, although it is not yet clear what triggers this inflammatory response and where it begins. Gut dysbiosis seems to be a likely candidate for the initiation of the systemic inflammation. The therapies in current use provide only symptomatic relief, but do not interfere with the disease progression. Nonetheless, animal models have shown promising results with therapies that target various vicious neuroinflammatory cascades. Translating these therapeutic strategies into clinical trials is still in its infancy, and a series of issues, such as the exact timing, identifying biomarkers able to identify Parkinson’s disease in early and pre-symptomatic stages, or the proper indications of genetic testing in the population at large, will need to be settled in future guidelines.

## 1. Introduction

Neurological disorders have escalated as the leading cause of disability worldwide and among them, Parkinson’s disease (PD), the second most common neurodegenerative disease after Alzheimer’s disease [1], has exhibited the fastest rate of growth in terms of prevalence, disability, and deaths [2]. Between 1990 and 2015, the number of persons living with PD doubled, exceeding 6 million [3], and is expected to double again by 2040 [4]. Additional factors, such as aging, declining smoking rates, increased industrialization, and use of pesticides and glues, could raise these numbers as high as 17 million [2].

The classical symptoms of PD include bradykinesia, rigidity, resting tremor, and postural instability in later stages [5], but a series of non-motor symptoms such as olfactory dysfunction, constipation, anxiety, sleep disorders, or depression may precede the motor symptoms by more than a decade [6,7,8]. Unfortunately, due to the lack of biomarkers that could enable an early diagnosis, PD diagnosis is currently based on clinical signs and can be confirmed post mortem with pathological studies which show significant loss of dopamine producing neurons in the substantia nigra pars compacta [9] and the accumulation of Lewy bodies in the neurons of the substantia nigra, dorsal nucleus of the vagus nerve, cerebral cortex, as well as the sympathetic ganglia or intestinal myenteric plexus [10]. These inclusions consist of abnormally folded α-synuclein which aggregate together with other proteins. However, Lewy bodies are not specific for PD, but can be found in other synucleinopathies, such as dementia with Lewy bodies or multiple system atrophy [11]. 

The etiology of PD is unknown, although the disease has been associated with several factors such as genetics [12], head trauma [13], air pollution [14], exposure to pesticides [15], prior infections [16], or alterations in the gut microbiota [17]. Nonetheless, research has provided convincing evidence of the involvement of oxidative stress, mitochondrial dysfunction, and impaired proteostasis in the pathogenesis of several neurodegenerative diseases, including PD [18,19,20,21,22]. In addition, neuroinflammation is increasingly recognized as having a significant contribution in neurodegeneration and may become a therapeutic target in the future [23,24]. 

The present review discusses the mechanisms and cascades through which neuroinflammation contributes to the onset and progression of PD. The references cited in this manuscript have been obtained from the PubMed and Google Scholar databases using as search criteria “Parkinson’s disease” AND “neuroinflammation”. We referenced full-text articles, experimental studies, and meta-analyses. No limits were set. 

## 2. Evidence of Neuroinflammation in Parkinson’s Disease

Human studies, animal studies, as well as in vitro studies point to the involvement of neuroinflammation in the pathogenesis of PD. 

### 2.1. Human Studies

In 1988, McGeer and coworkers provided the first neuropathological evidence of neuroinflammation in PD by describing large numbers of reactive microglial cells in the substantia nigra of patients in postmortem brain samples [25]. In the following years, similar studies described increased numbers of major histocompatibility complex (MHC) II positive cells in the putamen, hippocampus, transentorhinal, and cingulate cortex, as well as in the temporal cortex in PD brain samples compared to healthy controls [26]. 

These findings were confirmed by in vivo PET imaging studies [27], which showed increased binding of the [^11^C]-PK11195 radioligand, a ligand that binds to the translocator protein (TSPO) of activated microglia [28] in the midbrain and basal ganglia [29]. However, this ligand shows non-specific binding, short half-life, and reduced blood–brain barrier permeability, which is why new TSPOs have been developed and tested, such as [^18^F]-DPA714 or [^11^C]-PBR28 [28]. 

Moreover, studies performed on serum of patients revealed increased levels of a range of pro-inflammatory cytokines, such as TNF-α (tumor necrosis factor-α), interleukins IL-1β, IL-2, IL-10, or IL-17, as well as an increased number of T helper 1 cells, the authors also being able to link higher pro-inflammatory profiles with enhanced motor progression and cognitive decline [30,31]. 

### 2.2. Animal Studies

Animals are widely used to mimic human diseases. For PD, both transgenic models and sporadic ones are used, the latter using various toxins. 6-Hydroxydopamine (6-OHDA) shows a preferential uptake by dopamine and noradrenergic transporters, accumulates in the cytosol, and results in cell death without apoptotic characteristics via generation of reactive oxygen species (ROS) and quinones [32,33]. The dopaminergic neurotoxin 1-methyl-4-phenyl-1,2,3,6-tetrahydropyridine (MPTP), an analogue of the narcotic meperidine (Demerol), is the most commonly used toxin PD model. After crossing the blood–brain barrier, it is converted to 1-methyl-4-phenyl-2,3-dihydropyridium by monoamine oxidase B (MAO-B) in non-dopaminergic cells and then oxidizes to 1-methyl-4-phenylpyridinium (MPP^+^), being released into the extracellular space and taken up by dopamine transporters into dopaminergic neurons, where it binds to mitochondria and inhibits mitochondrial complex I of the electron transport chain (ETC) [34]. Paraquat (N,N-dimethyl-4–4-4-bypiridinium, PQ) is a herbicide structurally similar to MPP^+^ that enters the brain through a neutral amino acid carrier and inhibits complex I of the mitochondrial ETC, contributing to the generation of ROS which, in turn, oxidize proteins, lipids, DNA, and RNA. Rotenone, used as herbicide and insecticide, belongs to the rotenoid family. Delivered systemically, it causes degeneration of nigrostriatal dopaminergic neurons in the substantia nigra pars compacta along with aggregation of α-synuclein into Lewy bodies [35]. An alternative model is the lipopolysaccharide (LPS) model, whereby LPS is administered into the animal brain and induces dopaminergic neuronal loss in the substantia nigra [36]. 

In all these models, there was a robust microglial activation and increases in pro-inflammatory cytokines peaking before the dopaminergic neuronal loss [37,38]. 

Since a series of mutations have been identified as causing familial forms of PD, the development of animal models, from nematodes, to fruit fly and mammalian models, are crucial in basic pathophysiological research [39]. The many α-synuclein transgenic mouse models differ in the promoter and in the type of synuclein encoded (whether wild type or mutated α-synuclein). *LRRK2* (leucine-rich repeat kinase) knockout mouse models have been generated using conditional knockout strategies [40]. As for the autosomal recessive genes linked to PD, such as *Parkin*, *PINK1*, or *DJ-1*, knockout mice models do not exhibit the characteristic nigrostriatal pathology [39]. The rat brain offers a better representation of the human neurological system. Alternatives are bacterial artificial chromosome (BAC) models, which use large-size genomic DNA inserts, or viral-based models, in which the nigral delivery of engineered genes is achieved via adeno-associated viruses [41]. All these transgenic animal models of PD showed increased microglial activation [42] and increases in pro-inflammatory cytokines [43] as well.

### 2.3. In Vitro Studies

Cellular models are very helpful in studying specific cell types, and several cell lines have been developed for deciphering the pathophysiology of PD. They enable the study of cell–cell communication and interaction. However, they do not cover all the cell types present in the central nervous system [44]. Through the development of induced pluripotent stem cell-derived co-culture systems, researchers have the opportunity of evaluating the contribution of non-neuronal cells in neurodegeneration as well via inducing a variety of differentiated cells from PD patients to become midbrain dopaminergic neurons [45]. In particular, 3D cultures, such as brain organoids or spheres, allow for the modelling of multiple cellular interactions. Nonetheless, brain organoids have cells of neuroectodermal origin and so microglial cells, originating from the yolk sac, need to be added to the 3D culture [44]. A significant challenge is to maintain physiological distributions and ratios of the various cell types in the culture. In addition, mimicking the blood–brain barrier composition poses additional problems [44,46].

## 3. Microglia and Astrocytes in Parkinson’s Disease 

### 3.1. Microglia

Microglia, identified by Pío Del Río Hortega more than 100 years ago [44], are the resident macrophages of the brain and derive from a single mesodermal lineage, mesoblastic cells of the yolk sac infiltrating the nervous system in early stages of development and differentiating into microglia [47,48]. Depending on the anatomical region of the brain, microglia represent between 0.5% and 16% of all brain cells [49]. 

Aside from maintaining CNS homeostasis and mediating the innate immune response in the brain, microglia controls neurogenesis during CNS development by phagocytizing apoptotic cells in the subgranular zone [50], promoting neuronal survival by releasing insulin-like growth factor-1 (IGF-1) [51], eliminating redundant synapses through synaptic pruning [52], and regulating myelination by promoting oligodendrocyte survival and eliminating aberrant myelin membranes [53].

**Figure 1:**
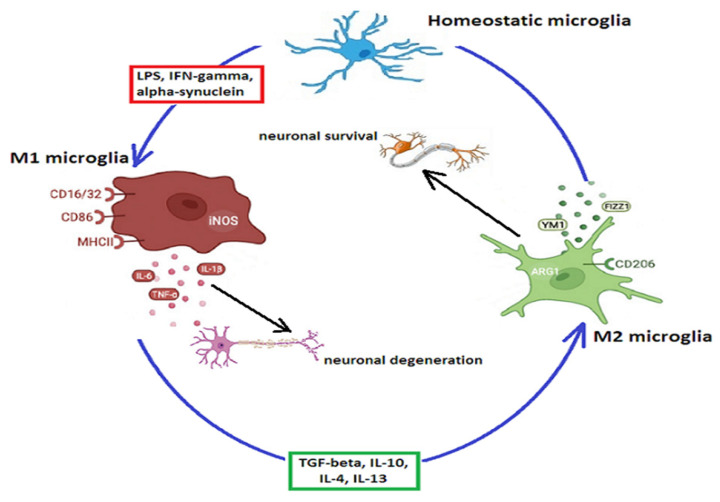
M1 and M2 microglial phenotypes. Normally, microglia exhibit the resting, homeostatic phenotype. Pathogens, such as LPS, α-synuclein, or other misfolded proteins, as well as interferon-γ, shift microglia toward the M1 pro-inflammatory phenotype, which produces ROS and pro-inflammatory cytokines such as IL-6, IL-1β, NOS, or TNF-α, with subsequent neuronal degeneration. Cytokines such as TGF-β, IL-10, IL-4, or IL-13 lead to the transition of the microglial M1 phenotype to the anti-inflammatory M2 phenotype, which promotes neuronal survival by secreting anti-inflammatory cytokines such as FIZZ1 or YM1. Abbreviations: Arg1—arginase-1; CD—mannose receptors (cluster of differentiation); FIZZ1—found in inflammatory zone protein 1; IFN—interferon; IL—interleukin; LPS—lipopolysaccharides; MHC—major histocompatibility complex molecules; NOS—nitric oxide synthase; TGF—tumor growth factor; TNF—tumor necrosis factor; Ym1—chitinase-3 like-3 (in rodents known as YM1).

In performing these complex functions, microglia shift from a homeostatic (ramified) state, in which they surf around to detect changes in the environment [54], to a reactive state, taking on an ameboid shape with extended soma, short processes, and modified surface receptors and secreting either pro-inflammatory cytokines and chemokines, known as the M1 phenotype, or anti-inflammatory factors which resolve the immune response and promote neuronal survival and regenerative processes—the M2 phenotype [23], as shown in Figure 1. 

### 3.2. Astrocytes

Astrocytes are the most abundant glial cells in the CNS, outnumbering neurons by about five times [55]. They participate in maintaining the integrity of the blood–brain barrier and regulate cerebral blood flow as well as neuronal activity [56]. Astrocytes also support neuronal functions by regulating glucose metabolism and maintaining the homeostasis of the neuronal environment [57], supplying energy to neurons through lactate [58], providing NADPH and glutamine to neurons [59], and they may even donate mitochondria to neurons [60]. Astrocytes form tripartite synapses with pre- and postsynaptic neurons and clear excess neurotransmitters [61] and potassium via the inward rectifier potassium channel Kir4.1 [62]. They regulate the extracellular water content via aquaporin-4 [63], and secrete a series of trophic factors, such as glial-derived neurotrophic factor (GDNF) or brain-derived neurotrophic factor (BDNF), to promote survival and differentiation of neurons [64]. 

Regarding the neurotransmitter re-uptake systems, the most extensively studied is glutamate clearance, which occurs via the excitatory amino acid transporter1/2 (EAAT-1/2) [65] in a Na^+^-dependent manner, followed by activation of the Na^+^K^+^-ATPase to restore the Na^+^ concentrations in the astrocytes. The necessary ATP derives from glycolysis followed by oxidative phosphorylation in the mitochondria [66]. Lactate produced during glycolysis is supplied to neurons [67], and the enhanced glycolysis associates an increase in flux to the pentose–phosphate pathway, elevating the ratio of intracellular equivalents of NADPH, thereby protecting against oxidative stress. Nonetheless, the flux to the pentose–phosphate pathway is regulated not only by glucose utilization, but also by transcription factors, among which the Kelch-like enoyl-CoA hydratase-associated protein 1 (Keap1)/nuclear erythroid 2 p45 subunit-related factor 2 (Nrf2) system has a crucial role. Nrf2 is normally bound to Keap1 in the cytosol and the complex is ubiquitinated and degraded by the proteasomal system preventing its transcriptional activity. ROS bind to the cysteine residue of Keap1 and change its conformation, promoting its dissociation from Nrf2, which translocates to the nucleus and binds to antioxidant response elements regulating the transcription of antioxidants. As for dopamine, three transporters are able to transport dopamine in a Na^+^-dependent manner: dopamine transporters (DATs), norepinephrine transporters (NETs), and serotonin transporters (SERTs) [68], but astrocytes surrounding dopaminergic synapses appear to take up dopamine via NETs and SERTs [61]. Nonetheless, dopamine exposure increases astroglial glucose consumption and enhances the pentose–phosphate pathway via the Keap1/Nrf2 system, protecting neurons against oxidative stress enhanced by monoamine metabolism or auto-oxidation [69]. 

However, recent studies have revealed a regional heterogeneity of astrocytes [70], with less potassium currents, interactions with neurons, and less gap junctions in striatal astrocytes [71], which may contribute to the selective vulnerability of dopaminergic neurons [72]. 

In response to infections, brain injuries, or neurodegeneration, astrocytes become reactive and change their morphology and gene expression, being able both to enhance brain injuries and to promote recovery of the injured CNS [73], depending on the brain injury model used [74]. LPS-reactive astrocytes upregulate genes for the complement cascade and cause neuronal demise and synapse loss in neurodegenerative diseases [75]. Nonetheless, astrocyte activation is entirely dependent on microglia, since pure in vitro astrocyte cultures, or LPS treatment in animals lacking microglia have failed to achieve astrocytic activation [76,77]. These neurotoxic reactive astrocytes, conventionally termed A1 astrocytes, exhibit a toxic gain of function and lose the ability to form functional synapses, leading to increased cell death among neurons co-cultured with A1 astrocytes [76]. 

### 3.3. Glial Activation Pathways

The M1 microglial phenotype is the body’s first line of defense in eliminating foreign pathogens, and inducing T cells to trigger an adaptive immune response [78]. M1 microglia produce pro-inflammatory cytokines, such as IL-1β, IL-6, IL-12, IL-17, IL-18, IL-23, tumor necrosis factor (TNF)-α and interferon (IFN)γ, and chemokines (CC-chemokine ligand 2-CCL2), exhibit major histocompatibility complex II (MHCII) antigens, and upregulate inducible nitric oxide synthase (iNOS), cyclooxygenase 2 (COX-2), CD86, ROS, and reactive nitrogen species [27].

IFNγ agonizes the IFNγ receptors 1 and 2 (IFNγR1 and IFNγR2), followed by phosphorylation and activation of Janus kinase (JAK) 1/2. Phosphorylated JAK 1/2 activates signal transducer and activator of transcription 1 (STAT1), which translocates to the nucleus, activating the transcription of genes encoding interferon regulatory factors, cytokines, and chemokines characteristically produced by the M1 phenotype [78]. 

**Figure 2:**
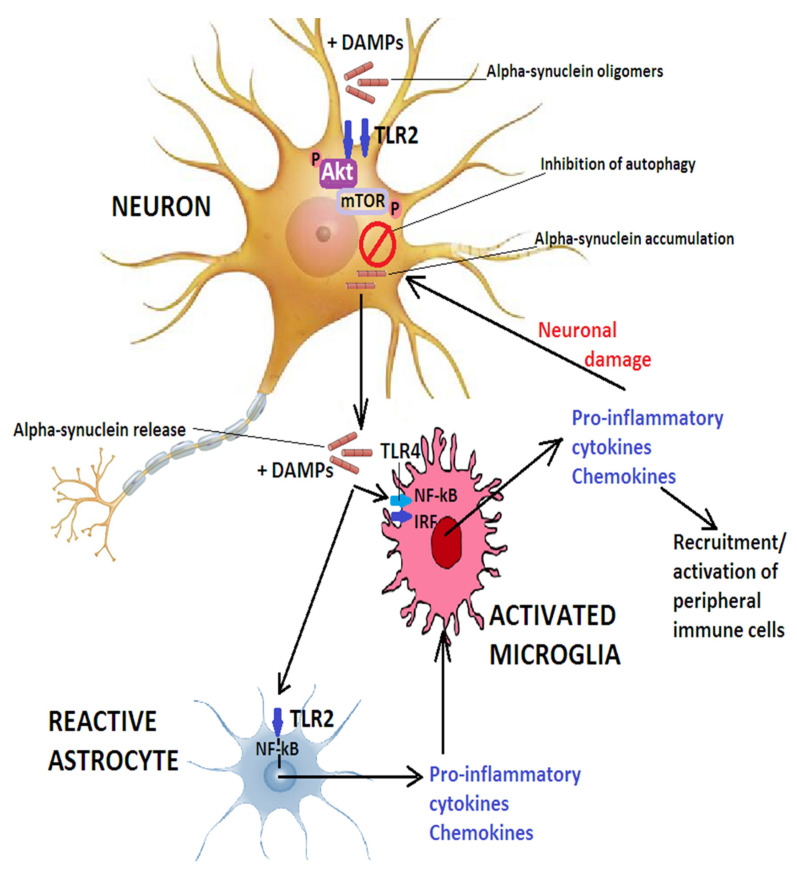
The role of TLR2 and TLR4 in PD. Oligomeric α-synuclein or other DAMPs activate neuronal TLR2 and inhibit autophagy via the Akt/mTOR pathway. This inhibits α-synuclein clearance and causes the release of the misfolded protein which, together with other DAMPs, activate microglia via TLR2 and TLR4 and leads to translocation of IRF and NF-κB to the nucleus and secretion of pro-inflammatory cytokines and chemokines that exacerbate neuronal damage and may additionally recruit peripheral immune cells to the CNS. A-synuclein released by neurons, together with other DAMPs, also triggers astrocyte activation via TLR2 and induces the production of pro-inflammatory mediators which can further contribute to microglial activation. Abbreviations: DAMPs—damage-associated molecular patterns; IRF—interferon regulatory factor; NF-κB—nuclear factor kappa light-chain-enhancer of activated B cells; TLR—toll-like receptor.

Toll-like receptors (TLRs) are highly expressed in microglia [79] and can bind proteins of viral origin or bacterial LPS, hyaluronic acid, or heat shock proteins as well as aggregated or misfolded α-synuclein released by neurons [80]. While TLR4-dependent activation of microglia involves the regulation of NF-κB, activator protein 1 (AP-1), and inflammasome pathways, oligomeric α-synuclein acting on TLR2 activates the NF-κB pathway [81], whereby NF-κB, normally bound to its inhibitor IκB in the cytoplasm, detaches from IκB and translocates to the nucleus where it promotes the transcription of target genes encoding for interleukin (IL)-6, COX2 and tumor necrosis factor (TNF)-α [82,83]. Figure 2 illustrates the role of TLRs in PD.

The mitogen-activated protein (MAP) kinases have also a significant role in M1 microglial activation. MAPKs are serine/threonine protein kinases with three main members: p38MAPK, c-Jun NH2-terminal kinase (JNK), and extracellular signal-regulated kinase (ERK1/2) [84]. JNK and ERK1/2 have important roles in microglial redox signaling [85], while p38 and JNK have been convincingly shown to be involved in LPS-dependent microglial activation resulting in enhanced transcription of AP-1 target genes and increased production of TNF-α, COX2, IL-6, and monocyte chemoattractant protein (MCP-1) [86]. Moreover, inactivation of p38MAPK prevents microglial activation through TNF-α signaling [87]. 

Mitochondrial dysfunction and oxidative stress, widely implicated in the pathogenesis of several neurodegenerative diseases [19], may contribute to neuroinflammation by activating inflammasomes, multiprotein oligomers formed by the inflammasome adaptor protein ASC, caspase-1, and components of the inflammasome such as nucleotide-binding oligomerization domain-pyrin domain-containing-3, -1 (NLRP3, NLRP1), NOD-like receptor (NLR) family CARD domain containing 3 (NLRC3), or absent in melanoma-2 (AIM2), which cleave pro-IL-1β to IL-1β or produce IL-18 [88]. In PD, NLRP3 inflammasome signaling has mainly been convincingly demonstrated, and ASC, the adaptor protein for the NLRP3 inflammasome, forms speck-like structures that may propagate the inflammasome in a prion-like fashion [89,90]. Pesticides, such as rotenone and tebufenpyrad, are able to induce mitochondrial dysfunction and activate the NLRP3 inflammasome, as well as altering lysosomal function [88], explaining the long-known epidemiological association between PD and pesticide exposure. Of the many mitochondrial quality-control mechanisms used by cells to limit mitochondrial damage, a locally directed repair pathway is interesting, namely the loading of damaged mitochondrial proteins and lipids into mitochondrial-derived vesicles (MDVs) and their transportation into the endolysosomal system for degradation [91]. The endosomal adaptor Toll-interacting protein (Tollip), in coordination with Parkin, facilitates the entry of MDVs carrying the translocase of the outer mitochondrial membrane, TOM20, into the endolysosomal system [92], while both Parkin and PINK1 are required for the formation of inner mitochondrial membrane-derived MDVs. In the lysosome, mitochondrial proteins are broken down, processed by the proteasome, and loaded onto major histocompatibility complex I (MHC I) molecules in the ER, followed by presentation at the plasma membrane. In addition, damaged mitochondria release mitochondrial DNA, able to activate the cGAS-STING pathway (cyclic GMP-AMP synthase–stimulator of interferon genes pathway). The dimeric cGAS protein receptor binds mitochondrial DNA and is activated, producing 2′3′-cGAMP that binds to STING, a protein located in the endoplasmic reticulum, and causes STING to dimerize and translocate to the Golgi apparatus, where it is phosphorylated by TANK-binding kinase 1 (TBK1) and binds to interferon regulating factor 3 (IRF3). Phosphorylated and activated IRF3 translocates to the nucleus and activates the transcription of interferons and pro-inflammatory cytokines [23,93]. PINK1 and Parkin suppress MDV formation and mitochondrial antigen presentation [94]. Moreover, Parkin and PINK1 knocked-out mice had a more prominent loss of dopaminergic neurons, which could be reverted in the absence of STING, highlighting the importance of the cGAS-STING pathway in PD [95]. Maintenance of the various Tollip-dependent pathways requires a delicate balance and in PD, elevated mitochondrial stress could lead to the saturation of Tollip function along the MDV route and compromise Tollip’s role in stabilizing STING. In addition, the sequestration of Parkin in an attempt to reduce mitochondrial stress alters Parkin’s capacity to regulate STING [91]. As such, MDVs play critical roles in the regulation of mitochondrial quality control and dynamics, which are severely impaired in PD pathogenesis and progression [96].

Damaged neurons, by releasing uracil nucleotides (uracil diphosphate and triphosphate, UDP and UTP) into the extracellular space, may supplementally contribute to microglial activation. UDP upregulates the expression of the metabotropic P2Y6 receptors via ERK1/2 phosphorylation and contributes to microglial activation, since knockdown of P2Y6 receptors was able to increase neuronal cell viability in an in vitro model [97]. In astrocytes, this pathway is more controversial, some studies reporting a protective effect against TNF-α-induced apoptosis via preventing the activation of caspases 3 and 8 [98], while other studies demonstrate a toxic effect, mediating NO production and astrocytic apoptosis [99]. 

Metabotropic glutamate receptors have also been widely studied in recent years in the context of neuroinflammation. According to their second messenger systems and specificity for agonists, these eight G-protein coupled receptors are classified into three groups: group I includes mGLuR1/5, group II includes mGLuR2/3, and group III includes mGLuR4/6-8 [100]. mGLuR5 has been shown to be involved in the pathogenesis of several neurodegenerative disorders, such as Alzheimer’s disease and Parkinson’s disease [101]. Alpha-synuclein hastens mGLuR5 degradation by promoting lysosomal degradation of the receptor, while activation of mGLuR5 inhibits α-synuclein-induced inflammation and signaling via the MAPK [102] and NF-κB pathway [103], as shown in both in vitro experiments as well as in animal models of PDF [104]. 

Protein kinase B (PKB), also known as the serine threonine kinase Akt, is activated by ligands binding to G-protein-coupled receptors or tyrosine kinase receptors. Through its SH2 domain, the regulatory component P85 interacts with the active receptor’s phosphorylated tyrosine residue, followed by the addition of the P110 catalytic subunit to create a fully functional PI3K enzyme. The second messenger phosphorylated from phosphatidylinositol (4,5)-disphosphate (PIP2) by P110 recruits inactive Akt from the cytoplasm to the cell membrane, followed by phosphorylation of threonine and serine residues on Akt [105]. One of the PI3K/Akt pathway’s downstream components is NF-κB [106]. In addition, phosphorylated Akt is significantly decreased in the substantia nigra—pars compacta—of PD patients [107] and activation of glycogen synthase kinase 3 (GSK-3) increases caspase-3 content in the dopaminergic neurons, resulting in their apoptosis [108]. Akt may inhibit GSK-3 activity by phosphorylating serine residues [109], thereby promoting neuronal survival in the substantia nigra.

Rho is a small GTPase belonging to the Ras superfamily, with a downstream effector protein, Rho kinase (ROCK), a serine/threonine kinase involved in cell morphology and polarity, gene expression, and cell division [110]. Inhibition of ROCK decreases the production of nitric oxide (NO), IL-1β, IL-6, and TNF-α, enhances the secretion of IL-10, and blocks the NF-κB pathway, promoting M2 microglial shift [111]. In addition, ROCK inhibition ameliorates mitochondrial dysfunction and ROS production [112], opening exciting new therapeutic opportunities in several neurodegenerative diseases [113]. 

The Notch signaling pathway controls a large number of cellular functions, including microglial polarization [114]. Activated Notch receptors are cleaved by γ-secretase and release an intracellular domain which translocates to the nucleus and acts as a co-activator, facilitating gene transcription [115]. Inhibition of the Notch 1 signaling pathway was able to reverse LPS-induced M1 microglial polarization promoting the M2 phenotype and decreasing pro-inflammatory cytokine production [116]. 

As mentioned above, activated microglia are able to convert astrocytes to a reactive, A1 phenotype, which secretes pro-inflammatory cytokines and becomes neurotoxic, no longer supporting the normal function of neurons [76].

The M2 microglial phenotype is involved in healing, slowing of inflammatory processes, and immunoregulation [48]. An immediate anti-inflammatory phase is activated concomitantly with the classical microglial activation [117], while in a later stage, an acquired deactivation further decreases the inflammatory response following phagocytosis of cells undergoing apoptosis or after exposure to anti-inflammatory cytokines such as IL-10 or tumor growth factor (TGF)-β [27]. M2 microglia, expressing the mannose receptor CD206 or the triggering receptor expressed on myeloid cells 2 receptor (TREM2), release arginase-1 (Arg-1), and growth factors like FIZZ1 (found in inflammatory zone protein), chitinase 3-like 3 (Ym1), PPAR (peroxisome proliferator-activated receptor), or IGF-1 which facilitate the deposition of extracellular matrix [54,118]. Actually, several activation states have been described. The M2a phenotype is induced by IL-4 or IL-13 and contributes to phagocytosis and tissue repair [78]. IL-4 stimulates JAK1 and 3 and activates STAT6 enhancing the transcription of CD206, suppressor of cytokine signaling 3 (SOCS3) and scavenger receptors (SRs). The M2b phenotype is involved in the recruitment of regulatory T cells and is promoted by the binding of ligands to TLRs and IL-1 receptors, which leads to the secretion of IL-10 and expression of CD86 and MHC-II. The M2c phenotype is induced by IL-10 and glucocorticoids. IL-10 binding to its receptors leads to activation of JAK1 and translocation of STAT3 to the nucleus, where it suppresses most of the M1-associated cytokine genes [119]. As opposed to peripheral immune cells, in which M1 polarization is terminal and which die during the inflammatory response, microglial cells can shift from the M1 to M2 phenotype following exposure to IL-10, beta interferons, glatiramer acetate, PPARγ agonists, or other molecules [78,120].

TREM2 is a key receptor linked to M2 microglial polarization [121]. It controls actin polymerization and the architecture of the cytoskeleton and increases ERK signaling through the adaptor protein, DNAX-activating protein 12 (DAP12, also known as tyrosine kinase binding protein—TYROBP, or killer cell-activating receptor-associated protein—KARAP) [122]. Of the many signaling pathways described, some promote, while others inhibit phagocytosis. To explain these contradictory effects, Turnbull and Colonna proposed an avidity-based model, in which a high-avidity interaction between a ligand and a DAP12-associated receptor would induce complete phosphorylation of DAP12 and recruitment of spleen tyrosine kinase (SYK), while a low-avidity interaction would incompletely phosphorylate DAP12 and lead to activation of the inhibitory phosphatase SH2-domain-containing protein tyrosine phosphatase 1 (SHP-1), followed by induction of inhibitory signaling [123]. In addition, TREM2 increases the expression of C-C motif chemokine receptor 7 on the surface of microglia and stimulates the migration toward CCR7 ligands [124], suppresses TLR signaling by inhibiting the MAPK pathway [125], and regulates the NF-κB signaling via activating the phosphoinositide 3-kinases/protein kinase B (PI3K/Akt) pathway [126]. 

Another important signaling pathway in mediating innate and adaptive immunity is the JAK/STAT pathway [127]. Although activation of the JAK2/STAT1 pathway enhances microglial M1 polarization by increasing the production of IL-1β, CXCL10, and TNF-α [128], activation of the JAK2/STAT6 pathway promotes the transition to an M2 phenotype because STAT6 controls the expression of Arg1, FIZZ1, YM1, and CD206 [129]. Due to the dual role of this pathway in microglial polarization, further research is required to fully describe its implication in PD as well as to identify possible therapeutic options. 

Many studies have demonstrated the transition of microglial phenotype M1 to M2 via the activation of AMP-activated protein kinase (AMPK) [130]. Inflammatory mediators increase intracellular calcium influx, leading to Ca^2+^ binding to calmodulin (CaM) and activating calcium/calmodulin-dependent protein kinase β, which in turn phosphorylates AMPKA [131]. Aging, a prominent risk factor for PD, associates a decline in mitochondrial activity and AMPK function [132].

Another family of receptors involved in the inhibition of expression of pro-inflammatory mediators in microglia and astrocytes is the nuclear receptor-related factor 1 (Nurr1) [133], belonging to the NR4A subfamily of orphan nuclear receptors (NRs) with important roles in regulating the expression of nigral dopaminergic neuronal genes such as tyrosine hydroxylase, dopamine transporters, vesicular monoamine transporter-2, and L-amino acid decarboxylase [134]. PD patients may exhibit decreased Nurr 1 expression, which is downregulated by α-synuclein via the NF-κB pathway [135], while mutations in Nurr1 can lead to familial forms of the disease [136]. In vitro studies showed that Nurr1 associates with NF-κB-p65 to target inflammatory gene promoters, recruits CoREST (RE1-silencing transcription factor) corepressor complex, and restores the expression of genes activated by NF-κB to basal levels [137]. Another receptor belonging to the same family is Nurr77, with low expression in the substantia nigra under basal conditions, but which is upregulated by chronic L-DOPA treatment (at least in a primate model of PD) [138], and which protects dopaminergic neurons from oxidative stress-mediated cell death and attenuates neuroinflammation [139]. 

Another class of nuclear receptors are the peroxisome proliferator-activated receptors (PPARs), with the three isoforms PPARα, PPARβ/δ, and PPARγ, which bind dietary lipids, eicosanoids, and non-steroidal anti-inflammatory drugs, such as ibuprofen or indomethacin [140]. They all exhibit anti-inflammatory effects by transrepression of NF-κB and by regulating oxidative stress pathways, but PPARγ is the most widely studied isoform [133]. 

### 3.4. Genetic Mutations Linked to Parkinson’s Disease and Neuroinflammation

The identified genetic mutations leading to familial forms of PD or to an earlier onset and rapid progression of the disease have deepened our knowledge of the pathogenic cascades.

#### 3.4.1. α-Synuclein and SNCA 

The characteristic Lewy bodies, abnormal intracytoplasmic inclusions of 5–25 μm in diameter, are composed mainly of α-synuclein that takes on a β-pleated sheet conformation [141]. The protein, encoded by the *SNCA* gene, belongs to the synuclein protein family and has three domains: an amino-terminal domain with 65 residues, a central, hydrophobic domain (residues 66–95), and an acidic carboxyl-terminal domain (residues 96–140) [142]. Under physiological conditions, it regulates the trafficking of synaptic vesicles in presynaptic terminals [72] and may also function as a chaperone [142], but in disease states, α-synuclein undergoes fibrillization and aggregation precipitated by ubiquitination and oxidative damage [142]. It is very likely that post-translational modifications, such as phosphorylation or nitration, increase the aggregation rate and neurotoxicity [143]. Once released into the extracellular space by damaged neurons, α-synuclein aggregates direct microglial migration toward the damaged neurons and binds to TLRs on microglia [80], functioning as damage-associated molecular patterns (DAMPs) and inducing robust microglial activation. Nitrated α-synuclein also activates peripheral leukocytes and accelerates neurodegeneration [144]. Moreover, fibrillar α-synuclein (but not monomeric protein) can activate the NLRP3 inflammasome, leading to the release of IL-1β, caspase-1, and ASC specks into the extracellular space [145]. 

A series of pathways regulating the persistent microglial activation are emerging. Fyn is a non-receptor tyrosine kinase acting as an upstream signaling molecule which, together with protein kinase C (PKC)-δ, influences MAP kinases and the NF-κB pro-inflammatory cascade in microglia [146]. Alpha-synuclein can also upregulate the expression of CXCL12 via TLR4/IκB-α/NF-κB signaling in microglia, which in turn promotes microglial migration by binding to the CXCR4 receptor [147]. Another protein, receptor-interacting protein kinase 1 (RIPK1), has been involved in microglial activation in LPS- and MPTP-induced animal models of PD [148]. Conversely, by binding to Fc gamma receptor IIB on microglia, α-synuclein can reduce microglial phagocytosis and impair the clearance of protein aggregates [149]. 

Point mutations, such as *A30P*, *A53T*, *H50Q*, *E46K*, or *G51D*, duplications, or triplications in the *SNCA* gene (*PARK1*) cause autosomal dominant forms of PD [44] with expression of mutant forms of α-synuclein that stimulate microglial cytokine release by activating MAPK pathways, including p38, ERK1/2, and JNK [150] earlier and more robustly [78]. Although *SNCA* shows a low expression in microglia [151], genetic variants could modulate *SNCA* expression in microglia and alter the immune profile and phagocytic ability of these cells [152]. 

In astrocytes, α-synuclein is expressed at lower levels as compared to neurons [153], and astrocytes accumulate the protein released by neurons, which leads to formation of inclusion bodies [154] and to the production of pro-inflammatory cytokines via the TLR4 pathway. In vitro, α-synuclein-activated microglia induced the reactive A1 phenotype in co-cultured astrocytes, converting them into neurotoxic cells [155]. 

#### 3.4.2. *PINK1* and *Parkin*

Loss-of-function mutations in *PINK1* and *PARK2* (coding for Parkin) lead to autosomal recessive familial forms of PD [156,157]. Both PINK1 (a serine threonine kinase) and Parkin (a ubiquitin E3 ligase) are involved in mitophagy and maintenance of mitochondrial quality control [19]. As such, altered mitochondrial quality control in neurons leads to neurodegeneration. However, the effects of these mutations in microglial biology are just beginning to be understood. It is likely that dysfunctional mitochondria generate high amounts of ROS and release mitochondrial DAMPs, shifting microglia toward a more inflammatory phenotype [158]. Parkin deficiency could enhance NLRP3 activation in microglia [159], while PINK1 may modulate cellular responses to IL-1β downstream of the inflammasome [160]. 

In addition, Parkin and PINK1 contribute to the repression of mitochondrial antigen presentation, whereby antigens are displayed on major histocompatibility complex proteins on the cell surface and detected by T lymphocytes, the externalization being mediated by mitochondria-derived vesicles [161]. In the absence of Parkin and PINK1, the production of these vesicles increased. In addition, aberrant antigen recognition may have causal relevance in PD, given the link of genetic variants in the human leukocyte antigen (HLA) system with the disease [162]. 

Experimental findings have confirmed the aforementioned impairments. *Parkin* knockout in BV2 microglia reduced microglial necroptosis, thereby prolonging inflammation and precluding the replacement of pro- with anti-inflammatory microglia [163], while *PINK1* knockout glial cultures showed increased nitric oxide and decreased anti-inflammatory IL-10 production [164]. In animal models, Parkin-deficient mice showed increased motor abnormalities and neurodegeneration in the substantia nigra in response to LPS injections [165]; meanwhile, upon transcriptomic analysis in PINK1 deficient mouse brains, an increased microglial inflammatory activity was demonstrated [166]. 

In astrocytes, Parkin regulates the expression of the inflammatory response in a nitric oxide-dependent manner [167] and Parkin-deficient astrocytes exhibited increased cytokine and decreased trophic factors’ release, augmenting neuronal vulnerability to neurotoxins [168]. Loss of PINK1 enhanced the astrocytic pro-inflammatory response and led to increased iNOS, NO, TNF-α, and IL-1β expression [164]. 

#### 3.4.3. Leucine-Rich Repeat Kinase 2 (*LRRK2*) and PD 

Both familial and sporadic forms of PD were associated with mutations in the *LRRK2/PARK8* gene, with incomplete and age-dependent penetrance [169]. LRRK2 is a serine/threonine kinase, PD-causing mutations enhancing its activity and contributing to neurodegeneration [170] by modulating inflammatory activity and oxidative stress in microglia exposed to fibrillar α-synuclein aggregates [171]. Animals harboring the most common *LRRK2* mutations linked to PD (G2019S and R1441C/G) manifested dysregulated transmission at dopaminergic and non-dopaminergic synapses similar to those observed in the prodromal phase of the disease and exhibited greater susceptibility to various parkinsonian toxins or stressors [172]. 

However, the involvement of microglial LRRK2 in PD pathogenesis is controversial. In mice, overexpressing the *R1441G LRRK2* mutation showed dopaminergic cell loss in response to systemic LPS delivery but the researchers failed to demonstrate *LRRK2* expression in microglia isolated from the experimental animals [173]. Furthermore, microglial LRRK2 mRNA or LRRK2 protein were not detected in anatomopathological studies [174]. These findings might be explained by the alterations in microglial phenotypes upon removal from the CNS environment [175]. 

Nonetheless, research has highlighted the involvement of LRRK2 in regulating microglial motility via focal adhesion kinase [176], microglial mitochondrial fission [177], and vesicle trafficking [178], suggesting that mutations could increase phagocytic function and lead to a more ameboid inflammatory microglial phenotype [179]. 

In astrocytes, LRRK2 is involved in the autophagy–lysosome pathway [180]. Inhibition of LRRK2 kinase induced mTOR-independent autophagy in astrocytes [181], while expression of mutations related to PD led to enlarged lysosomes and diminished lysosomal activity [182]. 

#### 3.4.4. *DJ-1* and Parkinson’s Disease

DJ-1, encoded by the *PARK7* gene, acts mainly as an oxidative stress sensor localized to mitochondria, where it increases the expression of two mitochondrial uncoupling proteins (UCP4 and UCP5), thereby decreasing the mitochondrial membrane potential and suppressing ROS production [183]. It regulates various transcription factors, such as Nrf2, PI3K/PKB, and p53, potentiating the production of endogenous antioxidants, and interacts with mitochondrial B cell lymphoma (Bcl)-XL protein as well as with inositol 1,4,5-trisphosphate (IP3) receptors in inhibiting apoptosis [183]. 

DJ-1 deficient microglia have elevated monoamine oxidase activity, which induces higher levels of intracellular ROS and nitric oxide and increases IL-1β and IL-6 secretion in response to dopamine exposure [184]. In addition, they also express higher levels of phosphorylated signal transducer and activator of transcription (STAT) 1 in response to IFNγ which, together with the attenuated interaction of phosphorylated STAT1 with Src-homology 2-domain containing tyrosine phosphatase-1 [185], leads to increased microglial neurotoxicity.

In astrocytes, the expression of DJ-1 is higher as compared to neurons [186] and regulates mitochondrial function, oxidative stress, as well as inflammatory response [72]. In an experimental setting, DJ-1 knockdown exacerbated rotenone-induced impairments of mitochondrial fission [187], led to increased nitrative stress [188], and to increased production of COX-2 and IL-6 after LPS treatment [189]. DJ-1 also associates with lipid rafts [190], cholesterol-enriched membrane domains with a crucial role in synaptic transmission, endo- and exocytosis, as well as signal transduction [191]. The astrocytic dysregulation of the inflammatory response appears to be related to TLR4 receptors, DJ-1 knockout astrocytes having impaired lipid raft assembly leading to impaired TLR4 endocytosis [192]. 

#### 3.4.5. Glucocerebrosidase (*GBA*) and Parkinson’s Disease

The glucocerebrosidase gene (*GBA*) codes for a lysosomal enzyme involved in glucocerebroside metabolism. Homozygous *GBA* mutations lead to Gaucher’s disease, while heterozygous mutations are recognized as risk factors for PD [193], increasing the risk for developing PD by 1.4- to 10-fold and decreasing the age of onset [194]. By reducing the enzymatic activity of glucocerebrosidase, the mutations impair lysosomal protein degradation and increase the exosomal release of α-synuclein [195], impairing the ability of microglia to clear cellular debris and molecules able to ignite inflammation such as α-synuclein. In addition, accumulation of glucocerebrosidase could activate complement and exacerbate microglial neurotoxicity [196]. Risk variants in the *CTSB* locus (coding for cathepsin B), by decreasing the expression of cathepsin B, may further modify the *GBA* mutation carrier’s risk for PD, in addition to age of onset [194]. 

*GBA* mutations in astrocytes cause abnormal α-synuclein inclusions [197], astroglial activation, and altered lysosomal cathepsin enzymatic activity [198] that alters proteostasis. In addition, the lysosomal dysfunction and TLR4-dependent inflammatory response could be normalized by inhibition of LRRK2 kinase activity, an observation that suggests functional cross-talk between LRRK2 kinase and GBA [199]. 

#### 3.4.6. Matrix Metalloproteinases 

Belonging to the family of extracellular membrane-bound or soluble endopeptidases, matrix metalloproteinases (MMPs) contribute to the remodeling of extracellular proteins [200]. Researchers have convincingly demonstrated the activation of MMPs, mainly MMP-3 and MMP-9, in various models of PD and pathological improvement achieved by suppressing the activity of these enzymes [201]. MMP-3 is activated in dopaminergic neurons by stress conditions and released into the extracellular space, where it can activate microglia, increase the TNF-α levels, and enhance the NF-κB signaling pathway [202]. 

## 4. The Adaptive Immune System in Parkinson’s Disease

The main cells of the adaptive immune system are T cells, and abnormalities in the function and activation states of these cells have been implicated in many diseases, neurodegenerative ones included. Both postmortem examinations in PD patients and animal models revealed significant T-cell infiltration in the brain and reduction in the T cells in the peripheral circulation [203]. 

The infiltrated T cells can be classified according to their cell surface markers into CD4^+^ and CD8^+^ T cells, the latter predominating in the substantia nigra. CD8^+^ T cells are activated by recognizing MHC I molecules and release lymphotoxins, such as granzyme and perforin, to kill target cells [204]. CD4+ T cells, in turn, were associated with increased motor dysfunction [205] and appear to enhance PD pathology by activating the Fas–Fas ligand-induced extrinsic apoptosis of dopaminergic neurons [206]. However, CD4^+^ cells can be further divided into proinflammatory Th1 and Th17 cells and anti-inflammatory Th2 and Treg cells (regulatory lymphocytes) [203]. It appears that Treg cells decrease neuroinflammation in the early stages of the disease, but later their action is gradually reduced via suppression of the γδT cells, which decrease in the peripheral blood of PD patients [207], leading to an immune imbalance. Th17 lymphocytes, on the other hand, have been found increased in the blood of PD patients and in postmortem brain samples [208], suggesting a role for this subtype of T cells in PD pathogenesis. Moreover, MHC II, a protein linked to the genetic risk for PD, mediates the recognition of antigens presented on the microglial surface by T cells and is critical for the activation of both innate and adaptive immune responses to α-synuclein in PD [209]. Peripheral T cells of patients with PD are able to recognize α-synuclein peptides [210,211]. 

Among the causes implicated in T-cell immune dysregulation is dopamine, which interacts with the dopamine D3 receptor expressed on their surface and promotes the differentiation of CD4^+^ T cells towards Th1 and Th17, while dopamine binding to the D1 receptor inhibits the function of Treg cells [212]. Moreover, α-synuclein promotes the differentiation toward Th1 and Th17 T-cell phenotypes and suppresses Treg function [213]. 

Activated microglia release TNF-α and IL-1β, which enhance the expression of adhesion molecules (intercellular adhesion molecule 1—ICAM1 and vascular cell adhesion molecule 1—VCAM1), promoting infiltration of peripheral immune cells into the brain parenchyma [214]. Moreover, chemokines released by activated M1 microglia, such as TNF-α, IL-1β, and IL-6 induce the differentiation of naïve T cells into Th17 cells by activating STAT3 and inhibiting the differentiation into Tregs [215,216]. In addition, chemokine (C-X-C motif) ligands (CXCL) 9, CXCL10, CXCL1, and CXCL16 released by M1 microglia bind to C-X-C motif receptors (CXCR) 3 and 6 on the surface of T cells [120], further promoting the differentiation of T cells into cytotoxic cells and enhancing T-cell-mediated inflammation [217]. In a vicious cascade, Th1 cells, by releasing IFN-γ and TNF-α, increase the expression of microglial TLR4 receptors and enhance their activation and the release of pro-inflammatory chemokines [218]. 

Research has also revealed important interactions between microglia and T cells which are neuroprotective and which could be modulated in the future to slow down the progression of PD. The anti-inflammatory cytokines IL-10, IL-4, IL-13, and TGF-β released by neuroprotective M2 microglia inhibit the production of pro-inflammatory cytokines [219] and IL-4 or TGF-β as well as CCL1, CCL17, CCL22, or CCL24 binding to CCR4 and CCR8 and promoting the differentiation of naïve T cells into Th2 and Tregs [120,203]. Further, Th2 and Tregs can induce the shift from M1 to M2 microglial phenotypes by enhancing the JAK/STAT6 pathway and decreasing the activity of the JAK/STAT3 pathway in microglia [203]. In addition, IL-4 and IL-13 induce the expression of IL-1 receptor agonist, thereby blocking the induction of microglial IL-1β [220]. 

The failure of maintaining a physiological balance between the pro- and anti-inflammatory action of microglia and infiltrating T cells may be explained by an age-related defective negative feedback of the M1 microglia caused by a dystrophic phenotype, featuring denuclearization and fragmentation of microglial processes [221], and leading to a decrease in receptor expression and cytokine secretion. In addition, the age-associated increase in inflammatory CNS environment [22] promotes the conversion of microglia to neurotoxic phenotypes and production of cytokines and chemokines that further promote neuroinflammation in a vicious cycle [23,222]. 

## 5. Gut Dysbiosis in Parkinson’s Disease

Despite the well-established association between neuroinflammation and neurodegeneration, the trigger of neuroinflammation is still unknown. Debris of degenerating neurons might be an activator for microglia, and neuromelanin has been shown to induce neuroinflammation in rat substantia nigra [223]; however, the finding that T cells from PD patients recognize specific α-synuclein epitopes [210] opens the interesting perspective of a trigger for neuroinflammation in PD outside the nervous system. 

**Figure 3:**
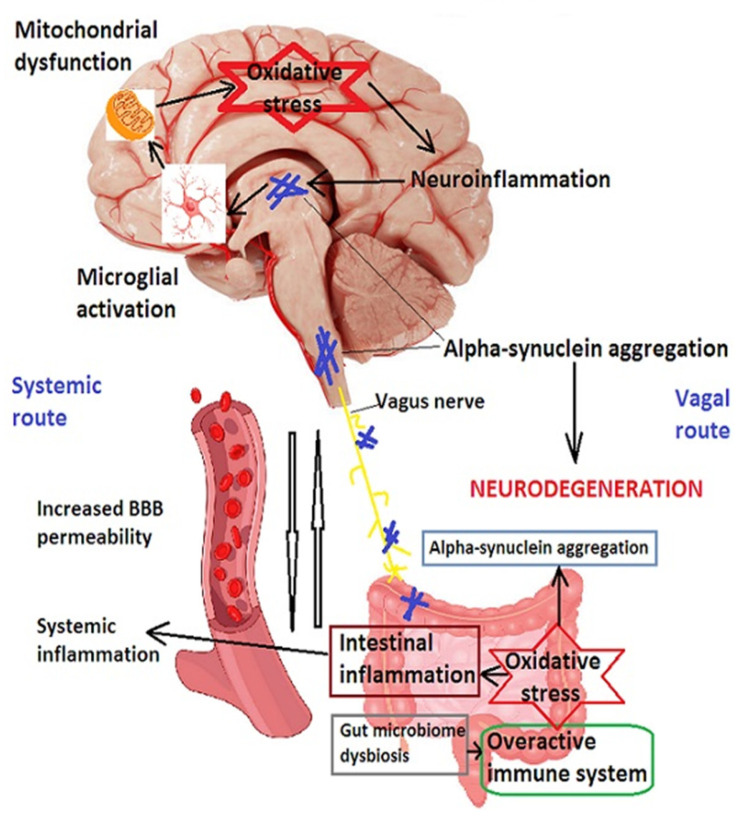
The gut–brain axis in Parkinson’s disease. Alterations in the gut microbiome lead to activation of the immune system and increase in oxidative stress, resulting in enhanced permeability of the intestinal epithelium, which allows bacterial products and α-synuclein to spread via systemic circulation and/or the vagal route from the enteric plexuses to the CNS, where they induce microglial activation, mitochondrial dysfunction, promote oxidative stress and neuroinflammation, potentiating α-synuclein aggregation, and resulting in neurodegeneration. BBB—blood–brain barrier.

Indeed, α-synuclein has been detected in cervical lymph nodes in animal models of the disease [144], and α-synuclein inclusions appear early in the enteric nervous system and dorsal nucleus of the vagus nerve as well as in the lower brainstem nuclei in patients who develop PD [224]. These findings suggest a spread of α-synuclein from the gut to the CNS via the sympathetic nervous system and the glossopharyngeal and vagus nerves [225,226]. Moreover, epidemiological findings suggest that patients who underwent truncal vagotomy are at decreased risk of developing PD [227], as are patients with a removed vermiform appendix [228]. In addition, abnormal brainstem pathology can be induced in rodents by injecting α-synuclein into gut tissue [229]. All these findings have led to the hypothesis that α-synuclein aggregation starts in the gut, where dysbiosis could lead to local inflammatory reactions and abnormal permeability of the intestinal epithelium, creating the premises for α-synuclein and bacterial products to spread to the central nervous system (CNS) via systemic circulation or the enteric sympathetic nervous system [230], as shown in Figure 3. The enteric nervous system, composed of the myenteric plexus (Auerbach’s plexus), submucosal plexus, and enteric glial cells, is a relay station between the gut microbiota and the CNS [231]. Enteric glial cells (EGCs) have numerous glial processes of different sizes and shapes, and markers of mature EGCs consist of GFAP, glutamine synthetase and brain fatty-acid binding protein [231]. 

Post-translational modifications of α-synuclein, such as C-terminal truncation by caspase-1 or cleavage by calpain, cathepsin, or MMPs, may render the protein more prone to misfolding [232]. The hypothesis is supported by the experimental proof of viral and bacterial infections being able to upregulate enteric α-synuclein [233] as well as by showing that repeated administration of an extracellular amyloid protein secreted by Escherichia coli (curli) to rats enhances neuronal deposition of α-synuclein in the gut and brain [234]. 

Studies of the intestinal microbiota have revealed interesting findings. This microbiota consists of a large number (a hundred trillion) of microorganisms located mainly in the lower gastro-intestinal tract, the composition of which depends on genetic background as well as on individual food habits [235]. In humans, the main phyla of the gut microbiota are *Firmicutes*, *Bacteroidetes*, *Actinobacteria*, *Proteobacteria*, *Fusobacteria* and *Verrumicrobia*, with *Firmicutes* and *Bacteroidetes* accounting for about 90% of the microbial population, being subject to variations depending on diet, environment, age, and intake of antibiotics [236]. In patients with PD, several changes at the family and genus level (rather than the phylum level) were reported, with an increase in phyla *Verrumicrobia* and *Actinobacteria* and a decrease in phyla *Firmicutes* and Bacteroidetes, a microbiome resembling the one found in patients with inflammatory bowel disease [237]. Table 1 provides an overview of the studies performed and their findings regarding the differences in gut microbiota between PD patients and controls. 

Many factors may lead to this dysbiosis. The increase in the genus *Lactobacillus* may be related to the high prevalence of constipation in PD patients, while the opposite may occur in patients with the diarrhea-type of irritable bowel syndrome [246]. Increases in lytic phages, such as *Lactococcus* phages, lead to a decrease in *Lactococcus* spp. and may be a result of dysbiosis or may be introduced with dairy products [245]. The “Western” diet, rich in sugar and fat, changes the proportion of Bacteroides and Firmicutes [247]. External toxins may additionally contribute to gut dysbiosis. Rotenone, widely used in pesticides and insecticides, has been linked to a significant decrease in the genus Bifidobacterium in a mouse model of PD [248]. 

Changes in intestinal microbiota increase the permeability of the colon through complex mechanisms. A deficit of butyrate leads to impairment of tight junctions between colonic enterocytes and alters the expression of claudin 1 and claudin 2 [249], while decreased levels of short chain fatty acids (SCFAs) produced by bacteria during fermentation of dietary fibers and prebiotics in the colon supplementally alter gastrointestinal motility [240]. EGCs and glial-derived neurotrophic factor have essential roles in maintaining the intestinal epithelial barrier [250]. The gut microbiota influences the function of the host enteric nervous system through a variety of signals including short chain fatty acids (SCFAs), metabolites of bile acids, and neuromediators such as GABA, tryptophan precursors and metabolites, or 5-hydroxytryptamine (5-HT or serotonin) [236]. The mechanisms by which this influence occurs are just beginning to be elucidated.

SCFAs, such as formic acid, butyric acid or propionic acid, are produced in food fermented by gut microbiota. Most clinical studies have shown a decreased production of SCFAs in PD [240]. Butyrate can increase the expression of tight junction claudins by activating the Akt/mTOR signaling pathway and downregulating the expression of TLR4 and of pro-inflammatory cytokines [251]. SCFAs have been shown to play key roles in the regulation of microglial maturation, morphology, and function [252] and in inhibiting the secretion of IL-1β, TNF, and monocyte chemoattractant protein-1 [253]. 

Bile acids are metabolized by gut microbiota to unconjugated secondary and tertiary bile acids in the intestine. While the intestinal microbiota can produce different types and quantities of bile acid derivatives, the latter influence the survival and growth of microbiota [236]. A neuroprotective effect has been demonstrated for tauroursodeoxycholic acid via regulation of JNK activity and activation of the Akt survival pathway [254], as well as by promoting Nrf2 activation [255]. Furthermore, by inhibiting the activation of astrocytes and microglia, tauroursodeoxycholic acid inhibits neuroinflammation [256]. 

Gut microbiota also affect the release of neurotransmitters in the intestine. The majority of serotonin in the body is synthesized by enterochromaffin cells and plays an important role in regulating intestinal peristalsis and epithelial secretion. The gut microbiota promotes serotonin secretion by releasing 5-HT and activating the 5-HT4 receptors [257]. In addition, microbiota also regulate the production of GABA [258]. 

As already mentioned, the CNS microglial homeostasis is strongly influenced by gut microbiota. Extensive defects and immature microglial phenotypes were described in germ-free mice, and eradication of the host microbiota caused significant modifications in the mouse microglial population [252]. In addition, LPS located on the outer membrane of Gram-negative bacteria may ignite an inflammatory process in the colonic wall, leading to enhanced expression of various cytokines, TLR-4 [259], allowing the entrance of bacterial products into the systemic circulation, and prime microglia by binding to TLR4, inducing apoptosis via enhanced expression of caspase-3, and increasing α-nitration and oligomerization, thereby contributing to neuroinflammation and dopaminergic neuronal loss [230]. In addition, intranasal injection of LPS could activate microglia in the olfactory bulb and substantia nigra through IL-1β/IL-1R1 signaling, increasing α-synuclein-positive cells, and reducing the number of dopaminergic cells in animal experiments [260], highlighting the role of peripheral inflammation in the pathogenesis of PD. By weakening the blood–brain barrier, promoting A1 astrocytic polarization, and increasing microglial activation, peripheral inflammation leads to excess production of pro-inflammatory cytokines (TNF-α, IL-1β, IL-6, or IFN-γ), with deleterious effects on dopaminergic neurons. 

## 6. Stress and Parkinson’s Disease

Stress was defined by Selye as a “general adaptation syndrome”. Regardless of the stimulus that causes the syndrome, humans mobilize physiological resources, including the brain, the autonomic system, and the neuroendocrine system in the stress response, although individual factors such as emotional stability, vulnerability, or coping style modulate this response together with environmental factors [261]. Among the systems activated during the stress response is the hypothalamic–pituitary–adrenal system, which results in the release of corticotropin releasing factor and adrenocorticotropic hormone, the latter further leading to production of glucocorticoids by the adrenal cortex [262]. 

Glucocorticoid receptors act as ligand-dependent transcription factors that regulate the expression of genes involved in the maintenance of cellular homeostasis [263]. Historically, glucocorticoids are viewed as strong anti-inflammatory hormones; thus, would be associated with inhibition of pro-inflammatory microglia. However, studies performed on both the peripheral and central nervous system showed that under specific conditions relating to the timing and duration of stress, glucocorticoids may promote CNS inflammation and activate microglia toward the M1 phenotype [264]. For example, if glucocorticoids are administered prior to a systemic LPS challenge, they upregulate IL-1β and TNF-α mRNA in the hippocampus [265]. Other models of stress applied to animals before an inflammatory challenge were accompanied by an increase in pro-inflammatory mediators and microglial proliferation in various areas of the CNS [266], as well as downregulation of CD200, the neuronal glycoprotein that maintains microglia in a quiescent state [267]. The NLRP3 inflammasome appears to have an important role in the pro-inflammatory action of glucocorticoids. The activation of the NLRP3 inflammasome requires two independent signals. The first signal is the activation of TLRs or nucleotide-binding oligomerization-domain protein 2 (NOD2) and NF-κB signaling. The activity of NF-κB is enhanced by glucocorticoids [268]. After synthesis of the NLRP3 inflammasome components, signal 2 induces the oligomerization and assemblage of these components, resulting in secretion of pro-IL-1β and caspase-1-dependent cleavage into IL-1β. Several studies have elegantly demonstrated the induction of NLRP3 expression and function by glucocorticoids [269,270]. In addition, the stress-induced release of DAMPs may contribute to priming of brain microglia [271]. 

In animal models, stress and high corticosterone levels augmented nigral neuronal loss and motor symptoms [272], which could be prevented by inhibiting the glucocorticoid receptors. 

In human patients, observational studies support the involvement of chronic and emotional stress in PD onset. During World War I, soldiers were affected by “shell-shock”(currently termed post-traumatic stress disorder) [273], which included various mental disorders and sometimes motor symptoms such as resting tremor, bradykinesia, and postural instability [274]. Chronic stress also appears to be related to more severe symptoms of PD, such as dyskinesias, bradyphrenia, or sleep disturbances [275], while the prevalence of depression is four times higher in PD patients than in the general population [276]. Moreover, during aging, the stress response becomes impaired, hyperactive, and returns less efficiently to homeostatic conditions [277], exposing the brain to high levels of glucocorticoids for longer periods of time. The dysfunctional hypothalamic–pituitary–adrenal axis associates dopaminergic cell loss and motor disability [278,279]. 

## 7. Anti-Inflammatory Therapeutic Strategies in Parkinson’s Disease

Based on solid evidence on the involvement of neuroinflammation in the onset and progression of PD, test began on a series of molecules with anti-inflammatory action in animal models and even in clinical trials.

### 7.1. Non-Steroidal Anti-Inflammatory Drugs (NSAIDS) and Minocycline

Most NSAIDS act as COX-1 and COX-2 inhibitors while also diminishing NO synthesis. While this approach exhibited neuroprotective effects in vitro and in animal MPTP models of PD [280], in human trials the reported results were contradictory and a Cochrane Collaboration study concluded that the existing evidence does not support the use of NSAIDS in the prevention of PD except for ibuprofen, which might reduce the risk of developing PD [281]. 

Minocycline is a tetracycline antibiotic with anti-inflammatory properties and encouraging results in animal models [282], but it failed to provide any improvement in PD symptoms when administered in a clinical trial setting [283]. Thus, counteracting general inflammation appears not to be beneficial, which is why novel strategies, aiming at selectively interacting with specific receptors and modulating the M1 to M2 phenotype shift, have been developed. Nonetheless, most of these molecules are in preclinical testing and only a few have completed clinical trials. 

### 7.2. Immunomodulators

Sagramostim, a granulocyte macrophage colony-stimulating factor acting as an immunomodulator that converts Th1 and Th17 into Treg and restores immune homeostasis, is already approved by the Food and Drug Administration (FDA) for the treatment of patients receiving bone-morrow transplantation or cancer therapy [284]. In a rodent PD model, sagramostim prevented the degeneration of dopaminergic neurons [285], while in a phase 1b, non-blinded, open-label clinical trial (NCT03790670) the molecule administered at 3 μg/kg/day was proven safe, increased the numbers of Tregs, but in terms of efficacy it just “did not worsen the MDS-UPDRS Part III scores” [286]. It is true that the trial was not powered to prove efficacy (including only five patients) and further trials are necessary to address this issue. 

Vasoactive intestinal peptide (VIP) suppresses the function of Th1 and Th17 and induces Th2 and Tregs [287] while also inhibiting microglial activation and expression of IL-1β and TNF-α [288]. The VIP receptor-2 peptide agonist LBT-3627 showed positive results in animal models [289], but no clinical trial has been carried out so far. 

A series of immunomodulatory drugs approved for the treatment of multiple sclerosis have also raised interest in the treatment of PD. Glatiramer acetate activates the microglial M2 phenotype [290] and protected dopaminergic neurons in a MPTP mouse model via recruitment of T lymphocytes in the CNS, inhibition of microglial activation, and upregulation of GDNF expression [291]. Dimethyl fumarate dampened the increase in IL-1β and the activity of COX-2 in a MPTP model of PD [292], while fingolimod acted by reducing the number of T lymphocytes invading the brain, inhibiting TNF-induced inflammatory genes in astrocytes, and protecting against dopaminergic neuronal degeneration in MPTP-induced animal PD models [293]. 

### 7.3. Targeting Pro-Inflammatory Cytokines and Receptors Involved in Activation of Neuroinflammation

While complete blocking of the TNF signaling pathway is detrimental, TNFR1-specific antibodies, such as ATROSAB, were able to shift microglial TNF signaling toward the anti-inflammatory and neuroprotective TNFR2 pathway in a chemical lesion of the nucleus magnocellularis. A similar effect was obtained with specific TNFR2 agonists such as the soluble EHD2-scTNFR2. In combination with ATROSAB, it protected cholinergic neurons and their cortical projections in the nucleus basalis magnocellularis chemical lesion [294].

TLR2 and TLR4 have been convincingly linked to PD. TLR2 levels were increased in postmortem brain samples of PD patients, the TLR2 agonist PAM3CSK4 increased the levels of endogenous α-synuclein in vitro and in transgenic mice, while blocking TLR2 receptors with antibodies enhanced α-synuclein clearance [295] and blocked neuron-to-neuron and neuron-to-astrocyte α-synuclein transmission which promoted NF-κB-dependent pro-inflammatory responses [296]. 

ROCK inhibitors, such as statins [297], Y-27632 [298], or fasudil [299], showed promising results in animal models of PD both by acting on neuroinflammatory pathways and by upregulating mitophagy. Simvastatin and lovastatin were evaluated in phase II clinical trials (NCT02787590 and NCT03242499, respectively), but the results were modest [300]. Nonetheless, a phase II trial with fasudil (NCT05931575) is planned but not yet recruiting [301]. 

Glucagon-like peptide 1 (GLP-1) is a peptide hormone secreted by intestinal cells, but GLP-1 receptors are also found in the brain. Activation of these receptors leads to the activation of protein kinase A and of the PI3K/Akt signaling pathway which regulates NF-κB, the master regulator of transcription of pro-inflammatory cytokines [302]. NLY01, a GLP-1 receptor agonist, could inhibit the conversion of pro-inflammatory astrocytes induced by microglia in a mouse PD model [155] and is tested in a phase II trial in patients with early PD (NCT04154072) [284]. Exenatide, with proven anti-inflammatory activity in animal models [303], has completed a phase II clinical trial (NCT01971242, EXENATIDE-PD) and has entered a phase II trial (NCT04232969; no longer actively recruiting and expected to be completed in 2024) [301]. Liraglutide, another GLP-1 mimetic with a longer half-life compared to exenatide, is currently being tested in a phase II trial (NCT02953665), while semaglutide, a synthetic analogue of GLP1, is also being tested in a phase II trial (NCT036559682) in idiopathic PD [301]. 

LRRK2 inhibitors modulated pro-inflammatory microglial signaling in rats [304] and attenuated neuroinflammation, gliosis, and cytotoxicity in both AD and PD mouse models [305]. However, LRRK2 is also abundantly expressed in kidneys, lungs, and immune cells, and inhibition of LRRK2 carries the risk of infections [306], but a phase I trial with DNL201, an LRRK2 inhibitor, raised no significant safety concerns [307]. 

### 7.4. Targeting the NLRP3 Inflammasome 

MCC950, a systemic NLRP3 inflammasome inhibitor, has been shown to suppress microglial activation, decrease dopaminergic neuronal degeneration, and diminish motor deficits in a mouse model of α-synuclein-mediated toxicity [308]. Similarly, inhibiting NLRP3 inflammasome activation with microRNA miR-7 proved protective both in vitro as well as in a MPTP mouse model of PD [309]. 

### 7.5. Immunotherapies Directed against α-Synuclein

Both active and passive immunization against α-synuclein are being evaluated in clinical trials following encouraging results from animal studies and successful approaches in Alzheimer’s disease.

#### 7.5.1. Active Immunization

Affiris has developed two vaccines to be used as active immunization against the C-terminal region of α-synuclein: PD01A and PD03A. Both molecules have completed phase 1 trials in multiple system atrophy [310] and in PD [311,312]. Both compounds triggered an antibody response and had a good safety profile, although PD01A performed better [313]. Another molecule targeting α-synuclein oligomers, UB-312, is currently in a phase 1 clinical trial [314]. 

#### 7.5.2. Passive Immunization

Passive immunization approaches with monoclonal antibodies have been evaluated with PRX002, an antibody directed against the C-terminal of α-synuclein, developed by Biosciences Limited, which demonstrated good tolerability and safety as well as peripheral α-synuclein binding by the antibody [315]. The findings supported a phase 2 study (PASADENA) in patients with early PD [316], and continued with a phase 2b study (PADOVA) that is currently recruiting patients [313]. Another monoclonal antibody developed by Biogen, BIIB054 or Cinpanemab, was shown to be safe and well-tolerated in the completed phase 1 trial [317], but the phase 2 trial was discontinued because primary and secondary outcome measures were not adequately met. Finally, a third molecule, developed by Astra-Zeneca, known as MEDI1341, and which targets monomeric and aggregated α-synuclein, has successfully completed a phase 1 clinical trial [318]. 

Table 2 provides an overview of the clinical trials targeting immunity and inflammation in PD patients.

### 7.6. Stem Cell and Cell-Based Therapeutic Strategies

Stem cell research is rapidly evolving in recent years, exploring its potential in the treatment of various diseases, neurodegenerative ones included [22,23,326]. Neural stem cells, or induced pluripotent stem cells, do have the potential of suppressing inflammation through paracrine signaling-based mechanisms [327]. Nonetheless, harvesting neural stem cells poses significant ethical issues, and an alternative would be to use induced pluripotent stem cells or mesenchymal stem cells, which integrate easily into host tissues and have the potential for trans-differentiation into dopaminergic neurons [328]. Unfortunately, the systemic administration of MSCs may lead to pulmonary thrombosis [329], whereas intrathecal or intracranial administration requires invasive procedures, and their beneficial effect may be transient [330]. 

Using extracellular vesicles reproduces the same paracrine signaling-mediated effects and circumvents potential side effects of stem cell delivery such as immunogenicity or tumorigenesis [331]. EVs released by mesenchymal stem cells recovered from the dental pulp of human exfoliated deciduous teeth have been actively pursued [332] and showed promising results in 6-OHDA rodent models. Glial-derived EVs, especially secreted by astrocytes from the ventral midbrain, rescued nigrostriatal projections in MPTP-treated mice [333]. Exosomes, the smallest extracellular vesicles, carry proteins, DNA and various RNA species, and are able to cross the blood–brain barrier, facilitating the delivery of selected molecules to the CNS [334]. Moreover, engineered small interfering RNAs (siRNAs), short hairpin RNAs (shRNAs), or shRNA minicircles, included in EVs can deliver anti-α-synuclein antibodies to reduce the aggregation of α-synuclein [335] or target the transcription of different genes involved in neuroinflammation. 

### 7.7. Targeting the Gut Microbiota

The gut microbiota composition can be manipulated with several approaches, such as probiotics, prebiotics, synbiotics, postbiotics, fecal microbiota transplantation, or dietary modifications. Probiotics are live microorganisms that can confer a health benefit on the host upon administration in sufficient amounts. Prebiotics are mainly non-digestible ingredients (fibers) that selectively stimulate the growth of some genera such as Bifidobacteria or Lactobacilli [336]. Synbiotics are a combination of prebiotics and probiotics, while postbiotics are compounds secreted by live bacteria or released after bacterial lysis that confer healthiness to the host (e.g., SCFAs, butyrate, propionate). In PD patients, probiotics administered for alleviating gastrointestinal dysfunctions also improved the UPDRS motor score and quality of life [337]. The beneficial effects of probiotics containing various bacteria have been reinforced by a large number of studies in animal PD models [338,339]. Prebiotics alone have not been evaluated in PD to date, although the decreased SCFA-producing bacteria in the PD microbiome could be corrected with prebiotic fibers and impact the modulation of inflammatory processes and intestinal permeability. Fecal microbiota transplant as a treatment strategy in PD is still in initial phases and used mainly for improving gastrointestinal symptoms like constipation or bloating, but reports on small studies highlight improvements also in the quality of sleep, anxiety, depression, as well as motor symptoms [340]. Currently, a phase 1 study (NCT05446168, BUTTER) is evaluating the safety of tributyrin supplementation of SCFAs in 20 patients, while another phase 1/2 trial (NCT05204641, EFFACE-PD) has enrolled patients by invitation to evaluate the safety and efficacy of fecal microbiota transfer [301]. 

Dietary modifications, favoring a high amount of fiber and polyphenol intake, is associated with a lower incidence of PD [341], while the Western diet, low in fiber, is correlated with increased incidence rates of PD and neurodegenerative disorders and may even exacerbate the severity of PD [342]. These observations are not surprising, since many phytochemicals (resveratrol, curcumin, quercetin, and others) have well-demonstrated effects on various pathways, many of them involved in the modulation of inflammation. Despite their low bioavailability [343], repeated administration during a healthy diet may confer sufficient anti-inflammatory effects of these various molecules. 

## 8. Future Perspectives

Despite significant breakthroughs made in recent years in our understanding of the pathogenesis of PD, currently available treatments rely on supplying the deficient dopamine to the brain or stimulating dopaminergic receptors, which may improve motor symptoms, but are hardly able to modify the course of the disease. One of the reasons is that diagnosis is usually made late, based on the motor symptoms of PD, which emerge when 50–70% of the nigral dopaminergic neurons are already lost [344], the disease has already evolved for at least a decade, and a series of vicious cycles have been ignited and are self-perpetuating. 

In our opinion, before entering a new era in the treatment of PD, several issues must be addressed:

1. Identifying reliable biomarkers could help in diagnosing PD in early stages, before the onset of motor symptoms. Several markers have been proposed and studied. First, low levels of fractalkine (CX3CL1) have been linked to the severity of PD [345]. Another possible neuroinflammation biomarker would be neurosin, a serine protease capable of hydrolyzing α-synuclein, that has been found to be decreased in the CSF of PD patients [346]. However, since inflammation levels are likely to fluctuate throughout time, longitudinal studies may be needed to adequately quantify neuroinflammation [347]. Neurofilaments and neuromelanin may help identify neurodegeneration [348], while markers of lysosomal dysfunction such as cathepsin and glucocerebrosidase (GCase) combined with α-synuclein aggregates in the CSF may allow a more precise diagnosis [349]. The usefulness of these biomarkers will need to be addressed by future guidelines.
2. The increasing identification of genetic mutations that increase the risk of PD comes with additional challenges and controversies [350]: (1) what resources are needed for clinical genetic testing and what is the cost–benefit ratio? (2) should testing vary based on ethnic background? (3) is genetic testing appropriate without further medical actions? Nonetheless, identifying individuals at risk for PD would allow for a more detailed panel of analyses that could be translated in early disease-modifying therapies with the aim of delaying the clinical onset of the disease.
3. Novel human and humanized models of PD using induced pluripotent stem cell technologies could overcome the differences between human and rodent microglia and ensure the increased success rates of preclinical to clinical translation of therapeutic strategies [28].

As for gut dysbiosis, whether probiotics and even fecal microbiota transplantation could contribute to slowing down of PD progression needs careful assessment in further studies [351].

Nonetheless, after being able to diagnose early PD, a multitargeted approach, addressing α-synuclein aggregation, neuroinflammation, promoting neuroprotection and autophagy, and with the judicious use of stem cells or exosomes, could significantly improve the quality of life of patients with PD. 

## Figures and Tables

**Table 1:** Studies on the composition of gut microbiota in Parkinson’s disease and their findings.

Study	Samples	Findings	
		Increased	Decreased
Scheperjans et al., 2015 [238]	72 PD patients and 72 controls	-*Lactobacillaceae* (phylum *Firmicutes*) -*Verrucomicrobiaceae* (phylum *Verrucomicrobia*) -*Bradyrhizobiaceae* (phylum *Proteobacteria*)	*Prevotellaceae* (phylum *Bacteroidetes*)
Hasegawa et al., 2015 [239]	52 PD patients and 36 controls	-*Lactobacillus* (phylum *Firmicutes*) -*Enterococcacecae* (phylum *Firmicutes*)	-*Clostridium coccoides* group (phylum *Firmicutes*) -*Bacteroides fragilis* (phylum *Bacteroidetes*)
Unger et al., 2016 [240]	34 PD patients and 34 controls	-*Bifidobacterium* (phylum *Actinobacteria*) -*Enterobacteriaceae* (phylum *Proteobacteria*)	-*Prevotellaceae* (phylum *Bacteroidetes*) -*Lactobacillaceae* and *Enterococcaceae* (phylum *Firmicutes*)
Bedarf et al., 2017 [241]	31 PD patients and 28 controls	*-Akkermansia* (phylum *Verrucomicrobia*) -phylum *Firmicutes* unclassified	-*Prevotellaceae* (phylum *Bacteroidetes*) -*Eubacterium* (phylum *Erysipelotrichaceae*)
Hill-Burns et al., 2017 [242]	212 PD patients and 136 controls	-*Akkermansia* (phylum *Verrucomicrobia*) -*Lactobacillus* (phylum *Firmicutes*) -*Bifidobacteriaceae* (phylum *Bifidobacterium*)	-*Lachnospiracea* (phylum *Firmicutes*)
Petrov et al., 2017 [243]	89 PD patients and 66 controls	-*Bifidobacterium* (phylum *Actinobacteria*) -*Christensenella*, *Lactobacillus* (phylum *Firmicutes*)	-*Faecalibacterium* (phylum *Firmicutes*) -*Bacteroides*, *Prevotella* (phylum *Bacteroidetes*)
Heintz-Buschart et al., 2018 [244]	76 PD patients and 78 controls	-*Akkermansia* (phylum *Verrucomicrobia)*	
Tetz et al., 2018 [245]	31 PD patients and 38 controls	Abundance of lytic *Lactococcus* phages	-*Prevotellaceae* -*Lachnospiraceae* -*Lactobacillaceae* -*Streptococcaceae*

**Table 2:** Clinical trials with immune-modulatory and anti-inflammatory therapies.

Drug Type	Drug Name	Clinical Trial Phase	Clinical Trial ID	Outcomes Reported	Reference
Granulocyte macrophage colony-stimulating factor	Sagramostim	Phase 1	NCT01882010	-no safety issues -modest improvements in UPDRS -part III scores	[319]
		Phase 1b	NCT03790670	3 μg/kg/day was better tolerated, MDS-UPDRS-part III scores did not worsen, increased numbers and function of Tregs	[286]
GLP-1 analogue	Exenatide	Phase 2	NCT01971242	Positive effects on off-medication motor scores	[320]
Tyrosine kinase inhibitor	Nilotinib	Phase 2	NCT02954978	-safe, +/− reduction in α-synuclein oligomers in the CSF	[321]
mAb targeting the carboxy-terminal epitope of α-synuclein	PRX002 Prasinezumab	Phase 1	NCT02157714 NCT02095171	Safe, reduced serum α-synuclein levels	[315]
		Phase 2	NCT03100149 (PASADENA)		[322]
		Phase 2	NCT04777331 (PADOVA)	Still recruiting	[301]
mAb targeting the amino-terminal epitope of α-synuclein	BIIB054 Cinpanemab	Phase 1	NCT02459886	Safe, complexes of the drug with α-synuclein were detected in plasma of patients	[317]
		Phase 2	NCT03318523 (SPARK)	-efficacy not different than for placebo	[323]
Antibody against monomeric and aggregated α-synuclein	MEDI1341	Phase 1	NCT03272165	Lowered extracellular α-synuclein in interstitial fluid and CSF	[318]
		Phase 1	NCT04449484	No results posted	[301]
Vaccine targeting the carboxy-terminus of α-synuclein	AFFITOPE PD01A	Phase 1	NCT04449484	No results posted	[301]
Vaccine targeting α-synuclein	AFFITOPE PD03A	Phase 1	NCT02267434	Well tolerated, antibodies toward vaccine components	[324]
Synthetic peptide-based vaccine targeting α-synuclein	UB-312	Phase 1	NCT04075318	Still recruiting	[325]
		Phase 1/2	NCT05634876	Not yet recruiting	[301]
